# Efficient Muscle Regeneration by Human PSC-Derived CD82^+^ ERBB3^+^ NGFR^+^ Skeletal Myogenic Progenitors

**DOI:** 10.3390/cells12030362

**Published:** 2023-01-18

**Authors:** Ning Xie, Sabrina N. Chu, Cassandra B. Schultz, Sunny S. K. Chan

**Affiliations:** 1 Department of Pediatrics, University of Minnesota, Minneapolis, MN 55455, USA; 2 Stem Cell Institute, University of Minnesota, Minneapolis, MN 55455, USA; 3 Lillehei Heart Institute, University of Minnesota, Minneapolis, MN 55455, USA

**Keywords:** pluripotent stem cells, myogenic differentiation, muscle stem cells, transplantation, cell therapy, muscular dystrophy

## Abstract

Differentiation of pluripotent stem cells (PSCs) is a promising approach to obtaining large quantities of skeletal myogenic progenitors for disease modeling and cell-based therapy. However, generating skeletal myogenic cells with high regenerative potential is still challenging. We recently reported that skeletal myogenic progenitors generated from mouse PSC-derived teratomas possess robust regenerative potency. We have also found that teratomas derived from human PSCs contain a skeletal myogenic population. Here, we showed that these human PSC-derived skeletal myogenic progenitors had exceptional engraftability. A combination of cell surface markers, CD82, ERBB3, and NGFR enabled efficient purification of skeletal myogenic progenitors. These cells expressed PAX7 and were able to differentiate into MHC+ multinucleated myotubes. We further discovered that these cells are expandable in vitro. Upon transplantation, the expanded cells formed new dystrophin^+^ fibers that reconstituted almost ¾ of the total muscle volume, and repopulated the muscle stem cell pool. Our study, therefore, demonstrates the possibility of producing large quantities of engraftable skeletal myogenic cells from human PSCs.

## 1. Introduction

Muscle stem cells are endogenous PAX7^+^ cells that are primarily responsible for skeletal muscle maintenance and regeneration [1,2]. Muscle stem cells possess tremendous in vivo regenerative potential, such that a single muscle stem cell is capable of giving rise to several hundred myofibers and reconstituting the muscle stem cell pool [3,4]. However, the scarcity of muscle stem cells (only 1–2% of mononuclear cells in muscle tissue [5]), diminishes their potential clinical use for cell therapies. Thus, alternative approaches to generating sufficient engraftable muscle stem cells are required for disease modeling or regenerative applications.

Considering their source availability, expansion capability, and differentiation potential, pluripotent stem cells (PSCs) have been an excellent candidate for generating scalable skeletal muscle progenitors [6,7]. Several protocols have been developed to differentiate PSCs into skeletal myogenic progenitors in vitro, either by overexpressing transcription factors such as PAX3 or PAX7 [8,9], or by monolayer differentiation employing various growth factors and small molecules [10,11]. Because these differentiation protocols often generate a mixture of different cell types [12], the use of reporters or cell surface markers is essential to enrich skeletal myogenic cells [13,14,15,16]. In addition, although the skeletal myogenic cells derived from these in vitro differentiation protocols are capable of forming multinucleated myotubes in vitro, they do not reliably engraft in vivo.

A recent study suggested teratomas as a promising platform for modeling multi-lineage development and tissue engineering [17]. Our group has recently described an efficient method to differentiate mouse PSCs into skeletal myogenic progenitors via teratoma formation. The skeletal myogenic progenitors derived in this way are functionally similar to endogenous satellite cells, in that 40,000 cells are capable of regenerating ~80% of total muscle volume [18]. Furthermore, these cells are readily expandable in vitro while retaining high engraftment potential [19]. We also found, by single-cell transcriptomics, that human PSC-derived teratomas harbor several skeletal myogenic populations that resemble embryonic to fetal skeletal myogenic development in humans [20]. In this study, we sought to determine whether these skeletal myogenic cells in human teratomas possess regeneration potential after transplantation. We discovered that the cell surface markers CD82, ERBB3, and NGFR are sufficient to identify the engraftable skeletal myogenic population in human PSC-derived teratomas.

## 2. Materials and Methods

### 2.1. Animals

All experiments involving animals were approved by the University of Minnesota Institutional Animal Care and Use Committee with AAALAC accreditation. Experiments were performed on both male and female 3–4-month-old NSG-mdx^4Cv^ mice [21].

### 2.2. Pluripotent Stem Cell Culture

EGFP-labeled H1 human ESCs (WiCell WA01, Madison, WI, USA) were generated by inserting EGFP into the AAVS1 safe harbor locus as described previously [22]. H1 ESCs were cultured in TeSR-E8 medium (StemCell Technologies #05990, Vancouver, BC, Canada) on plates coated with Cultrex (R&D Systems #3434-010-02, Minneapolis, MN, USA). When cell confluency reached 50%, cells were dissociated with Accutase (Innovative Cell Technologies #AT-104, San Diego, CA, USA) and passaged.

### 2.3. Teratoma Formation

NSG-mdx^4Cv^ mice were anesthetized by a cocktail of ketamine (150 mg/kg, i.p., Akorn #NDC:59399-114-10, Lake Forest, IL, USA) and xylazine (10 mg/kg, i.p., Akorn #NDC:59399-111-50). For cell preparation, undifferentiated H1 ESCs (1 million in TeSR-E8 medium) were mixed with Cultrex at a 1:1 ratio (total 20 μL). The cell–Cultrex mixture was subsequently implanted via a 28 G insulin syringe (Covidien #8881600004, Mansfield, MA, USA), into the tibialis anterior (TA) muscles.

### 2.4. Teratoma Harvest

At 8 weeks, teratomas were harvested from the host animals and minced into small pieces (~2 mm). The pieces were then digested in 2 mg/mL Collagenase II (Gibco #17101-015, Gaithersburg, MD, USA) dissolved in Dulbecco’s Minimum Essential Medium/High Glucose (HyClone #SH30243.01, Logan, UT, USA) with 1% penicillin/streptomycin (P/S) (Life Technologies #15140-122, Grand Island, NY, USA) at 37 °C with constant shaking at 250 rpm. After 30 min, digested tissues were filtered through 40 μm cell strainers and washed with rinsing buffer (Ham’s/F-10 medium (Caisson Labs #HFL01, Smithfield, UT, USA), 1% HEPES buffer solution (Caisson Labs #HOL06), 10% horse serum (HyClone #SH30074.03), and 1% P/S (first-pass). A second 30-minute round of digestion was performed for undigested tissues (second-pass). Cells from the first-pass and the second-pass were subsequently combined and resuspended in FACS buffer (0.2% FBS (Gemini Bio-Products #100-119, West Sacramento, CA, USA) in PBS (HyClone #SH30256.01)) for FACS.

### 2.5. Cell Isolation from Transplanted TA Muscles

Transplanted TA muscles were removed from NSG-mdx^4Cv^ mice and then mechanically chopped into small pieces. Chopped tissues were incubated in primary digestion buffer consisting of DMEM/high glucose, 2 mg/mL collagenase II, and 1% P/S on a shaker at 250 rpm, at 37 °C for 75 min. Primary digestion was stopped by adding rinsing buffer, and tissue spun down at 1500 rpm for 5 min at 4 °C. The tissues were further digested in secondary digestion buffer consisting of rinsing buffer supplemented with 0.1 mg/mL collagenase II and 0.5 mg/mL dispase (Gibco, Cat#17105-041) on a shaker at 250 rpm, at 37 °C for 30 min. Digested tissues were vortexed, drawn, and released into a 5 mL syringe with a 16-gauge needle 4 times, and then with an 18-gauge needle 4 times. Dissociated tissues were filtered through 40 μm cell strainers, spun down, then washed with rinsing buffer and resuspended in FACS buffer on ice. Cells were processed for flow cytometry analysis of GFP^+^ cells.

### 2.6. Cell Transplantation

Cells were injected into the tibialis anterior (TA) muscles of NSG-mdx^4Cv^ mice for transplantation. Two days prior to cell injections, recipients were anesthetized with ketamine (150 mg/kg, i.p., Akorn #NDC:59399-114-10, Lake Forest, IL, USA) and xylazine (10 mg/kg, i.p., Akorn #NDC:59399-111-50), and left hindlimbs were exposed to 1200 cGy X-ray irradiation. One day before cell injections, 15 μL of cardiotoxin (10 μM, Latoxan #L8102, France) was injected into the irradiated muscles to induce muscle injury. On the day of transplantation, 100,000 cells in SkGM-2 medium (Lonza #CC-3245, Basel, Switzerland) with Y27632 (10 nM, APExBio #A3008, Houston, TX, USA) were injected into TA muscles using a Hamilton syringe. Freshly isolated 8EN cells were transplanted immediately after FACS sorting, and passage 3 8EN cells were transplanted immediately after cell dissociation from cell culture plates. The cell numbers reported for transplantation were not corrected for apoptosis. Grafted TA muscles were harvested at 4 months after transplantation and processed for immunostaining to evaluate engraftment, or harvested at 5 days after transplantation and processed for fluorescence-activated cell sorting (FACS) analysis to evaluate cell survival.

### 2.7. Fluorescence-Activated Cell Sorting (FACS)

Teratoma cells were stained with fluorophore-conjugated antibodies in FACS buffer for 30 min on ice. Propidium iodide (PI, 1 μg/mL, Sigma #P4170, Saint Louis, MO, USA) was added to the FACS buffer, and only live cells (PI–) were counted. A BD FACS AriaII machine, equipped with the FACSDiva software (BD Biosciences, San Diego, CA, USA), was used for cell sorting. FlowJo (FLOWJO LLC, Ashland, OR, USA) was used to generate FACS plots. The following antibodies (at 0.5 μL per million cells) were used for FACS: APC anti-CD82 (R&D Systems #FAB4616A, RRID:AB_2076404), PE anti-ERBB3 (BioLegend #304706, RRID:AB_2099569), and PECy7 anti-NGFR (BD Biosciences Cat# 562122, RRID:AB_10894762).

### 2.8. Apoptosis Assay

To quantify apoptotic cells, the FITC Annexin V Apoptosis Detection Kit with 7-AAD (BioLegend Inc., San Diego, CA, USA) was used in accordance with manufacturer’s instructions. Freshly isolated 8EN cells were assayed immediately after sorting into SkGM-2 cell culture medium. Passage 3 8EN cells were detached from tissue culture dishes using Accutase for 3 min, dissociated into single cells, resuspended in SkGM-2 cell culture medium, and then assayed. Fluorescence analysis was performed using BD FACS AriaII cytometer. A total of 10,000 cells were recorded for each sample and analyzed using FlowJo. Cells that were positive for 7-AAD and/or Annexin V were considered apoptotic.

### 2.9. Skeletal Myogenic Cell Culture

Human teratoma-derived skeletal myogenic cells were plated on 0.1% gelatin-coated wells and cultured in SkGM-2 medium (Lonza #CC-3245, Switzerland). Cells were passaged every 5–7 days when they reached 80% to 90% confluency. Cells were washed with PBS, then incubated with Accutase at 37 °C for 3 min to dissociate them for passaging. The cell number was counted at each passage.

### 2.10. In Vitro Skeletal Myogenic Differentiation

Skeletal myogenic progenitors were differentiated into myotubes at 80% to 90% confluency. The medium was switched to N2 medium (DMEM/F12, 1% ITS-A (Gibco #51300-044), 1% N2 (Gibco #17502-048), 1% Glutamax (Life Technologies #SCR006), 1% penicillin/streptomycin (Life Technologies #15140-122)) supplemented with 10 μM SB-431542 (APExBIO #A8249) for 4 days. Cells at the end of differentiation were subjected to immunostaining with a myosin heavy chain (MHC), MYOD, or PAX7.

### 2.11. Immunostaining on Cultured Cells

At room temperature, cell cultures were fixed with 4% paraformaldehyde (PFA) (Sigma #P6148) for 20 min, permeabilized with 0.3% Triton X-100 (Sigma #X100) for 30 min, and blocked with 3% bovine serum albumin (BSA) (Thermo Fisher Scientific #BP1605-100) for an hour. Cells were then incubated with primary antibodies (diluted in 3% BSA) overnight at 4 °C, followed by secondary antibodies (diluted in 3% BSA) for an hour at room temperature. DAPI staining was used to identify nuclei. The following primary antibodies were used: rabbit anti-MYOD1 at 1:500, Santa Cruz Biotechnology #sc-304, RRID:AB_631992, Dallas, TX; mouse anti-PAX7 at 1:10, DSHB #PAX7, RRID:AB_528428; mouse anti-MHC at 1:20, DSHB #MF-20, RRID:AB_2147781, mouse anti-MHC-I (1:100, DSHB #BA-D5; RRID:AB_2235587), mouse anti-MHC-IIa (1:100, DSHB #SC-71; RRID:AB_2147165), mouse anti-embryonic MHC (1:100, DSHB #F1.652; RRID:AB_528358), and mouse anti-neonatal MHC (1:100, DSHB #N3.36; RRID: AB_528380). Secondary antibodies used were goat anti-mouse (IgG1, IgG2b) Alexa Fluor 555, goat anti-mouse (IgG1, IgG2b) Alexa Fluor 647, and goat anti-rabbit Alexa Fluor 647 (all from Life Technologies). Images were taken with a Zeiss AxioObserver Z1 inverted microscope equipped with an AxioCamMR3 camera. The ZEN software (Zeiss) was used for image capture and processing.

### 2.12. Immunostaining on Muscle Sections

TA muscles were harvested for analysis 4 months after cell transplantation. The harvested TA muscles were embedded in optimal cutting temperature (OCT) solution (Scigen #4586, Gardena, CA, USA), snap-frozen in liquid nitrogen-chilled 2-methylbutane (Sigma #320404), and stored at −80 °C. Muscle sections were cut at 10 μm on a Leica CM3050 S cryostat (Leica Microsystems, Buffalo Grove, IL, USA) and collected every other 250 μm across the TA muscle. Sections with the largest cross-sectional areas of the whole TA were used for fiber engraftment evaluation. Sections were rehydrated with PBS, permeabilized with 0.3% Triton X-100 for 30 min, and blocked with 3% BSA for 1 h at room temperature. Primary antibodies were incubated overnight at 4 °C, followed by secondary antibodies for 1 h at room temperature, and counterstained with DAPI. Slides were mounted with Immu-Mount (Thermo Scientific #9990402) and proceeded to image capture as described above. Images showing whole TA muscles were captured in a tile mode and stitched together using the Zen software. The primary antibodies used were rabbit anti-DYSTROPHIN (1:250, Abcam #ab15277; RRID:AB_301813), mouse anti-Lamin A/C (1:500, ThermoFisher Scientific #MA3-1000; RRID: AB_325377), mouse anti-MHC-I (1:100, DSHB #BA-D5; RRID:AB_2235587), mouse anti-MHC-IIa (1:100, DSHB #SC-71; RRID:AB_2147165), mouse anti-PAX7 (1:10, DSHB #PAX7; RRID:AB_528428), rabbit anti-laminin (1:500, Sigma #L9393; RRID:AB_477163), and Alexa Fluor 555 anti-α-bungarotoxin (1:100, Invitrogen #B35451; RRID:AB_2617152). The secondary antibodies used were goat anti-rabbit Alexa Fluor 488, goat anti-mouse Alexa Fluor 555, goat anti-mouse IgG1 Alexa Fluor 555, and goat anti-mouse IgG2b Alexa Fluor 647.

### 2.13. Myofiber Engraftment Area Measurement

Sections with the largest cross-sectional areas of the whole TA were used for myofiber engraftment evaluation. DYSTROPHIN or laminin staining was used to determine the cross-sectional area of muscle fibers. Myofiber engraftment is defined as the total cross-sectional area of DYSTROPHIN^+^ fibers over the total cross-sectional area of the whole TA section.

### 2.14. Quantitative RT-PCR

An RNeasy Mini Kit (QIAGEN #74106, Valencia, CA, USA) was used to extract the total RNA. A Verso cDNA Synthesis Kit (Thermo Scientific #AB1453A, Pittsburgh, PA, USA) was used to perform genomic DNA removal and reverse transcription to obtain cDNA. cDNA was then mixed with Premix Ex Taq (probe qPCR) master mix (Clontech #RR39WR, Mountain View, CA, USA) and Taqman probes (Applied Biosystems, Carlsbad, CA, USA). A QuantStudio 6 Flex Real-Time PCR System (Applied Biosystems) was used for quantitative polymerase chain reaction (qPCR). The 2^−ΔΔCt^ method, which indicates the fold change in gene expression normalized to the housekeeping gene *GAPDH*, was used for data analysis. The following Taqman probes were used: *GAPDH* Hs99999905_m1, *PAX7* Hs00242962_m1, and *MYOD1* Hs00159528_m1.

### 2.15. Software

FACS analysis data acquisition was performed in FACSDiva v6.1.3 (BD Biosciences, San Diego, CA, USA) and analyzed in FlowJo v7.6.3 (FlowJo LLC, Ashland, OR, USA). Immunostaining images were acquired using ZEN v2.3 pro software (Zeiss, Jena, Germany). Myofiber area measurements were performed using ImageJ software (U. S. National Institutes of Health, Bethesda, MD, USA).

### 2.16. Statistical Analysis

Data are presented as mean ± SEM. All statistical analyses were performed using GraphPad Prism v6.07 (GraphPad Software, La Jolla, CA, USA). Significance was calculated using two-tailed unpaired Student’s t tests for comparison between the two groups, or one-way ANOVA with Tukey’s post hoc test for comparison among three or more groups. Differences are considered to be statistically significant at the *p* < 0.05 level: * *p* < 0.05, ** *p* < 0.01, and *** *p* < 0.001.

## 3. Results

### 3.1. Human Teratoma-Derived Skeletal Myogenic Progenitors Are CD82^+^ ERBB3^+^ NGFR^+^

In our previous work, we demonstrated that teratomas derived from mouse PSCs are a rich source of skeletal myogenic progenitors with exceptional engraftment potential and in vitro expandability [18,19]. We further showed that this teratoma formation strategy is also applicable to human PSCs for developing the skeletal myogenic lineage [20]. In the current study, we evaluated whether skeletal myogenic progenitors generated from human PSC-derived teratomas were able to engraft and regenerate damaged muscles in vivo.

We generated teratomas using EGFP-labeled human H1 ESCs, and 8 weeks later, we isolated the CD82^+^ ERBB3^+^ skeletal myogenic population by FACS as previously described. We then transplanted 100,000 of these freshly isolated CD82^+^ ERBB3^+^ cells to the tibialis anterior (TA) muscles of NSG-mdx^4Cv^ mice [21]. The NSG-mdx^4Cv^ mice were both immunocompromised and dystrophin-deficient, thus allowing allogenic cell transplantations and easy identification of donor cell-derived dystrophin^+^ fibers. The TA muscles were irradiated and cardiotoxin-injured prior to cell transplantation to enhance muscle regeneration [23]. Transplanted muscles were harvested 3 months later for engraftment evaluation. We readily observed human lamin A/C^+^ cells in the transplanted muscles, indicating that the human donor cells engrafted. However, the engrafted cells failed to develop into muscle fibers (0 donor-derived human lamin A/C^+^ dystrophin^+^ fibers were observed from >20 transplantations). We reasoned that the CD82^+^ ERBB3^+^ cell population found in human teratomas might have contained non-skeletal myogenic cell types that were inhibitory to donor cell contribution to new fiber formation in vivo. We decided to search for additional surface markers for further purification of the skeletal myogenic lineage.

**Figure 1:**
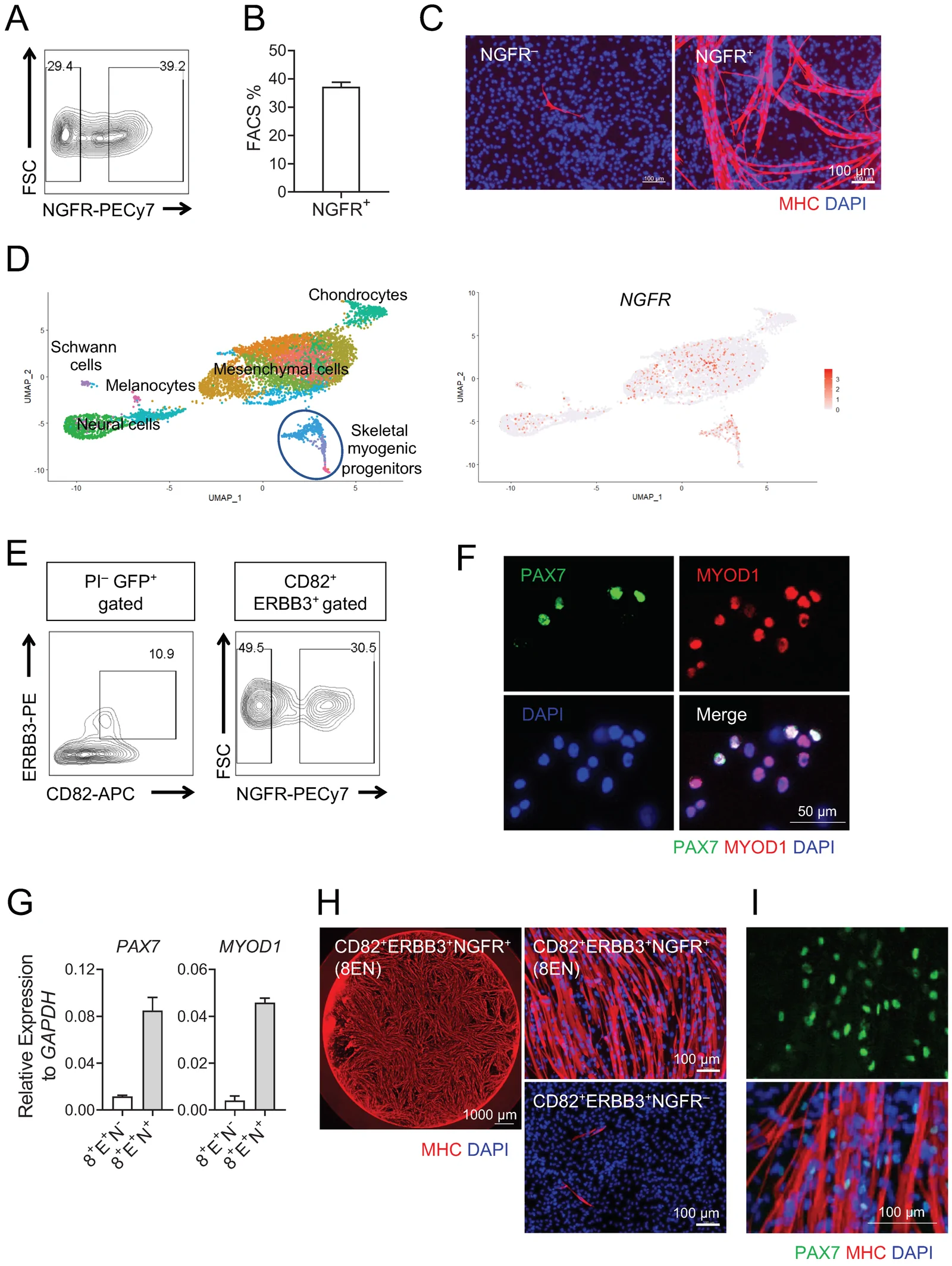
CD82, ERBB3, and NGFR mark the skeletal myogenic population in human H1 ESC-derived teratomas. Human H1 ESC-derived teratomas contained a NGFR^+^ cell population (mean ± SEM, 3 biological replicates): (**A**) FACS analysis and (**B**) quantification. (**C**) MHC^+^ myotubes were predominantly found in the NGFR^+^ cell population (scale bar = 100 μm). (**D**) Single-cell RNA-seq analysis of H1 ESC-derived teratomas (from [20]) revealed that NGFR was expressed in many different cell types, including skeletal myogenic cells. (**E**) FACS analysis indicated the presence of CD82^+^ ERBB3^+^ NGFR^+^ cells in human teratomas. (**F**) Human teratoma-derived CD82^+^ ERBB3^+^ NGFR^+^ cells expressed PAX7 and/or MYOD1 (scale bar = 50 μm). (**G**) RT-qPCR showed that *PAX7* and *MYOD1* expression was higher in CD82^+^ ERBB3^+^ NGFR^+^ cells than in CD82^+^ ERBB3^+^ NGFR^–^ cells. Data are shown as mean ± SEM from 3 biological replicates. ** *p* <0.01, *** *p* <0.001. (**H**) Differentiation of CD82^+^ ERBB3^+^ NGFR^+^ cells into MHC^+^ myotubes (scale bar = 1000 μm left; 100 μm right). (**I**) PAX7^+^ cells were found along with MHC^+^ myotubes in differentiated CD82^+^ ERBB3^+^ NGFR^+^ culture (scale bar = 100 μm). 8EN: CD82^+^ ERBB3^+^ NGFR^+^; MHC: myosin heavy chain.

We discovered that human teratoma-derived skeletal myogenic progenitors were predominately found in the NGFR^+^ cell population (Figure 1A–C). The NGFR^+^ cell population was likely to include cell types other than skeletal myogenic, as some NGFR^+^ cells were unable to develop into myoblasts/myotubes even under strong skeletal myogenic cues (Figure 1C). This as somewhat expected, given that in our previous single-cell RNA-seq analysis of human teratomas, we did not detect NGFR as a standalone skeletal myogenic surface marker, but rather found NGFR to be present in diverse cell populations (Figure 1D) [20]. Nevertheless, when combined with CD82 and ERBB3, the use of NGFR further purified the skeletal myogenic population in human teratomas (Figure 1E–H). Among CD82^+^ ERBB3^+^ cells, a NGFR^+^ fraction was distinctly observable (Figure 1E). While CD82^+^ ERBB3^+^ NGFR^–^ cells were minimally skeletal myogenic, CD82^+^ ERBB3^+^ NGFR^+^ cells (heretofore known as 8EN cells) expressed the skeletal myogenic transcription factors PAX7 and MYOD1 (Figure 1F–G) and were able to develop into myosin heavy chain^+^ (MHC^+^) myotubes upon differentiation (Figure 1H). Interestingly, we observed many PAX7^+^ cells residing along with MHC^+^ myotubes in the differentiation culture (Figure 1I), resembling a “reserve cell” population commonly found in in vitro cultures of endogenous muscle stem cells obtained from adult muscles [24].

### 3.2. Expanded CD82^+^ ERBB3^+^ NGFR^+^ Skeletal Myogenic Progenitors Express CD56 and Are Capable of Differentiating into Large Multinucleated Myotubes

**Figure 2:**
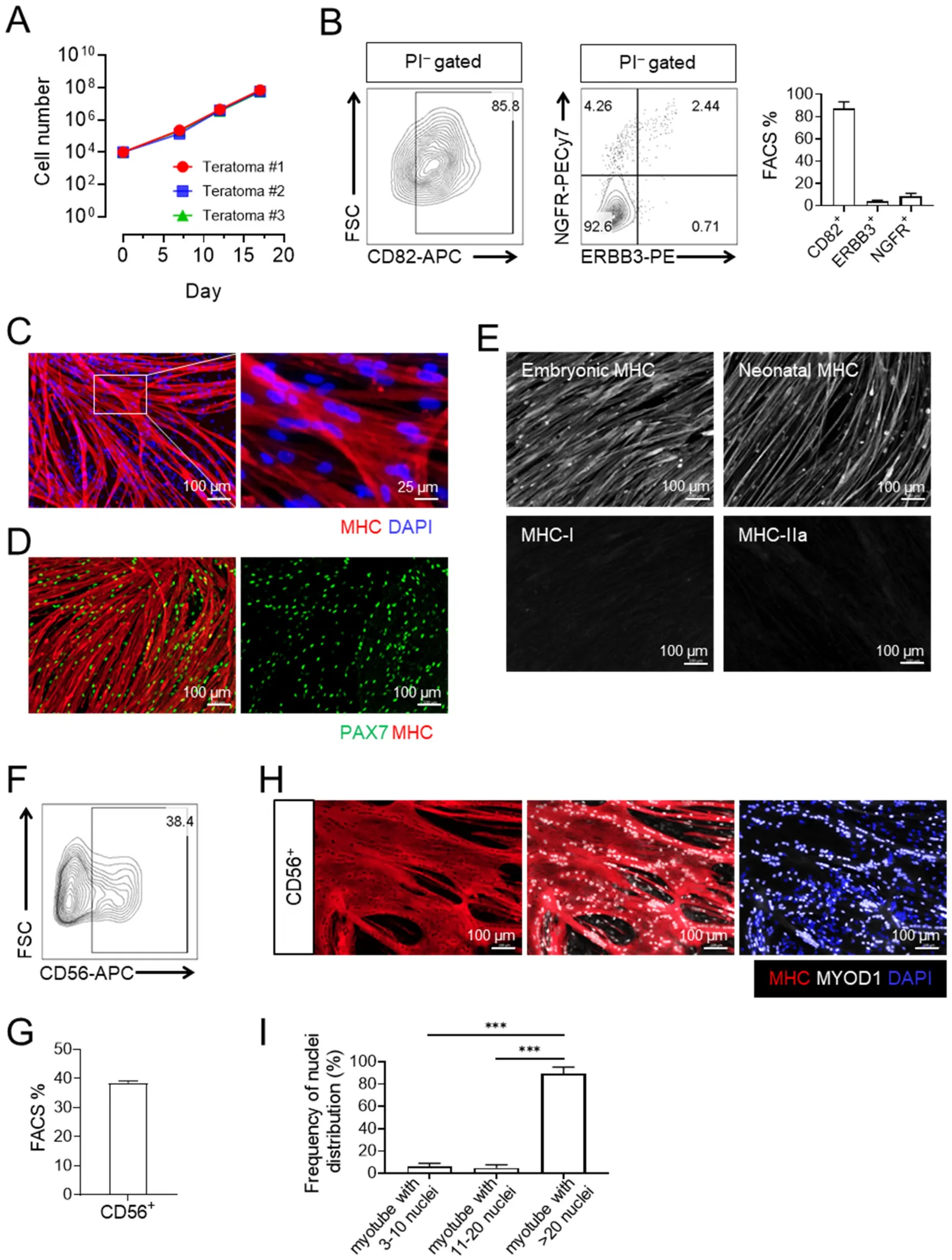
8EN skeletal myogenic progenitors develop into CD56^+^ muscle stem cell-like cells in vitro. (**A**) Growth curves of 8EN cells from 3 different human H1 ESC-derived teratomas. (**B**) FACS analysis and quantification of passage 3 8EN cells. Data are shown as mean ± SEM from 3 biological replicates. (**C**,**D**) Upon differentiation, passage 3 8EN cells developed into (**C**) MHC^+^ myotubes (scale bar = 100 μm left, 25 μm right) and (**D**) PAX7^+^ “reserve cells” (scale bar = 100 μm). (**E**) Myotubes generated from passage 3 8EN cells expressed embryonic and neonatal isoforms of MHC, but not the adult isoforms such as MHC-I and MHC-IIa (scale bar = 100 μm). (**F**) FACS analysis of passage 3 8EN cells showing the emergence of a CD56^+^ cell population. (**G**,**H**) Passage 3 8EN-derived CD56^+^ cells fused into large multinucleated (>20 nuclei) MHC^+^ myotubes (**G**, scale bar = 100 μm) and quantification of nuclei distribution (**I**). Data are shown as mean ± SEM from 3 biological replicates. *** *p* < 0.001. 8EN: CD82^+^ ERBB3^+^ NGFR^+^; MHC: myosin heavy chain.

We have previously shown that skeletal myogenic progenitors generated from mouse PSC-derived teratomas were expandable while maintaining their skeletal myogenic properties [19]. We next examined whether their human teratoma-derived counterparts behaved similarly. We found that 8EN cells grew steadily over 3 passages, expanding by ~4 orders of magnitude in 17 days (Figure 2A). For all three passages, cultured 8EN cells remained mostly CD82^+^, but the expression of ERBB3 and NGFR diminished (Figure 2B and Figure S1). At passage 3, 8EN cells remained strongly skeletal myogenic, and upon differentiation, were capable of developing into multinucleated MHC^+^ myotubes with a PAX7^+^ “reserve cell” population (Figure 2C,D). Furthermore, myotubes derived from passage 3 8EN cells expressed embryonic MHC and neonatal MHC, but not adult myosin isoforms such as MHC-I and MHC-IIa (Figure 2E).

Interestingly, we readily observed a CD56^+^ cell population emerging in passaged 8EN cells (Figure 2F,G). Remarkably, these 8EN-derived CD56^+^ cells had a high propensity to fuse together upon subsequent differentiation. The MHC^+^ myotubes thus produced were large, and consistently composed of 20+ nuclei (Figure 2H,I). These changes in expression of surface markers recapitulate human skeletal myogenic development, whereas ERBB3^+^ NGFR^+^ embryonic/fetal skeletal myogenic progenitors give rise to an adult CD56^+^ CD82^+^ muscle stem cell population [13,25,26].

### 3.3. Passaged CD82^+^ ERBB3^+^ NGFR^+^ Skeletal Myogenic Progenitors Improved Survivability

**Figure 3:**
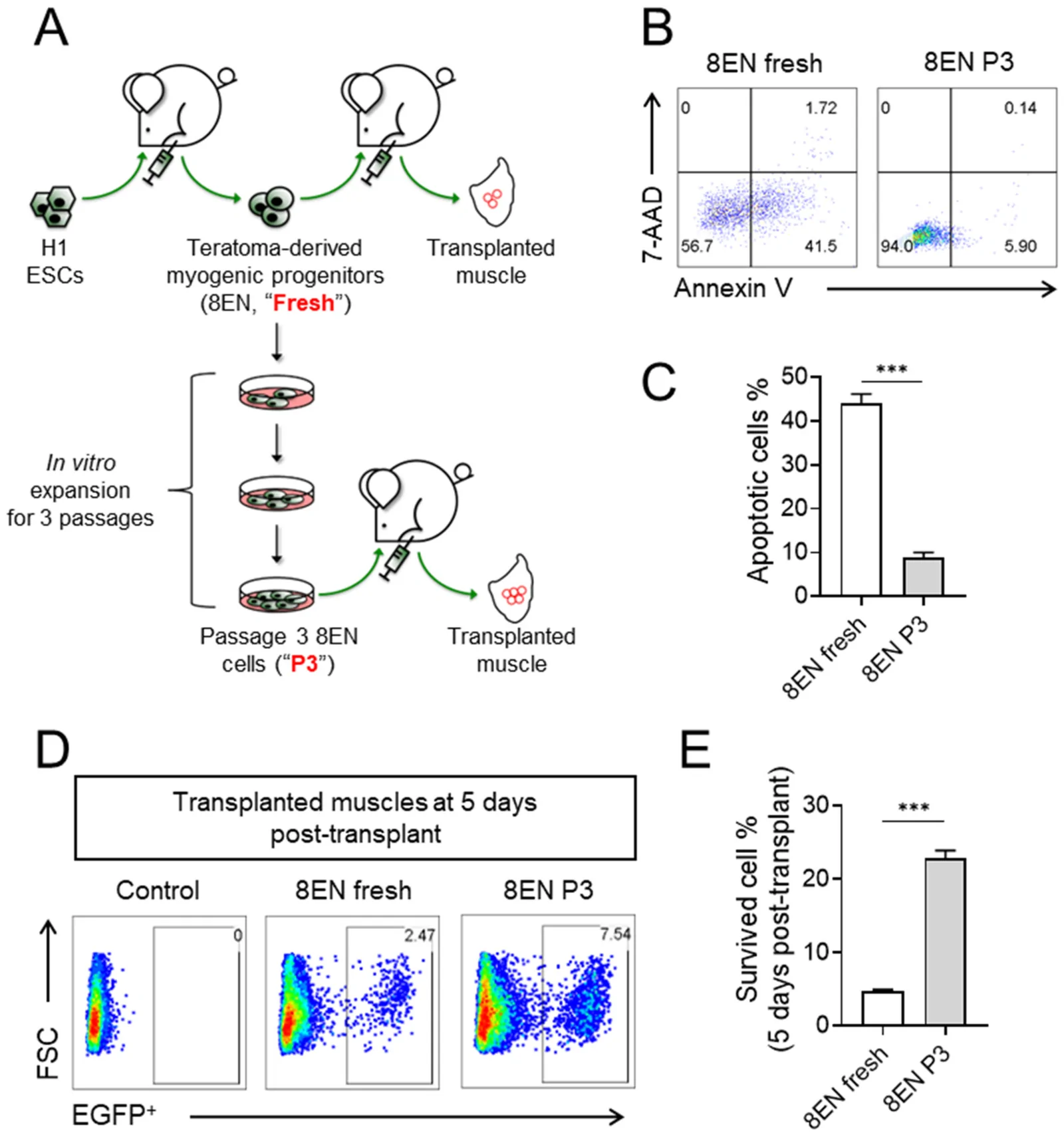
Passaged 8EN skeletal myogenic progenitors improved cell survival in vitro and in vivo. (**A**) Schematic of transplantation of 8EN cells produced from human H1 ESC-derived teratomas. (**B**) FACS analysis and (**C**) quantification (mean ± SEM, 4 biological replicates, *** *p* < 0.001) of apoptosis: cells expressing 7-AAD and/or Annexin V were considered apoptotic. (**D**) FACS analysis and (**E**) quantification (mean ± SEM, 4 biological replicates, *** *p* < 0.001) of EGFP^+^ donor cells 5 days after transplantation. The total mononuclear fraction in the transplanted TA muscles was analyzed in (**D**), and the number of EGFP^+^ donor cells was counted. The percentage of survived cells (**E**) was calculated as a ratio of the number of EGFP^+^ donor cells 5 days after transplantation to the number of injected cells (100,000 cells). 8EN: CD82^+^ ERBB3^+^ NGFR^+^.

We next investigated whether human teratoma-derived 8EN cells engraft and contribute to the skeletal myogenic lineage in vivo using transplantation assays (Figure 3A). We transplanted 100,000 8EN cells, freshly isolated from H1 ESC-derived teratomas, to the irradiated and cardiotoxin-injured TA muscles of NSG-mdx^4Cv^ mice and evaluated engraftment 3 months later. We observed very few donor-derived dystrophin^+^ fibers (<10% of total muscle volume), and some donor cells failed to develop into fibers (human lamin A/C^+^ dystrophin^–^). We wondered whether the poor engraftment was related to poor cell survival after transplantation. Indeed, when we performed 7-AAD/Annexin V staining on fresh 8EN cells immediately after their isolation by FACS, we found that >50% of them were apoptotic (i.e., Annexin V^+^) (Figure 3B,C, left). Unsurprisingly, freshly-isolated 8EN cells also did not survive well in vivo, as only ~5% (~4700 survived out of 100,000 injected) remained in the muscles 5 days after transplantation (Figure 3D,E, left). On the other hand, passaged 8EN cells were less apoptotic in vitro and showed better survival in vivo. Passaged 8EN cells had minimal 7-AAD or Annexin V expression (Figure 3B,C, right and Figure S2), and their retention rate in the transplanted muscles was ~3-fold that of their freshly-isolated counterparts 5 days after transplantation (Figure 3D,E, right).

### 3.4. Passaged CD82^+^ ERBB3^+^ NGFR^+^ Skeletal Myogenic Progenitors Regenerate Muscles in DMD Model Mice upon Transplantation

**Figure 4:**
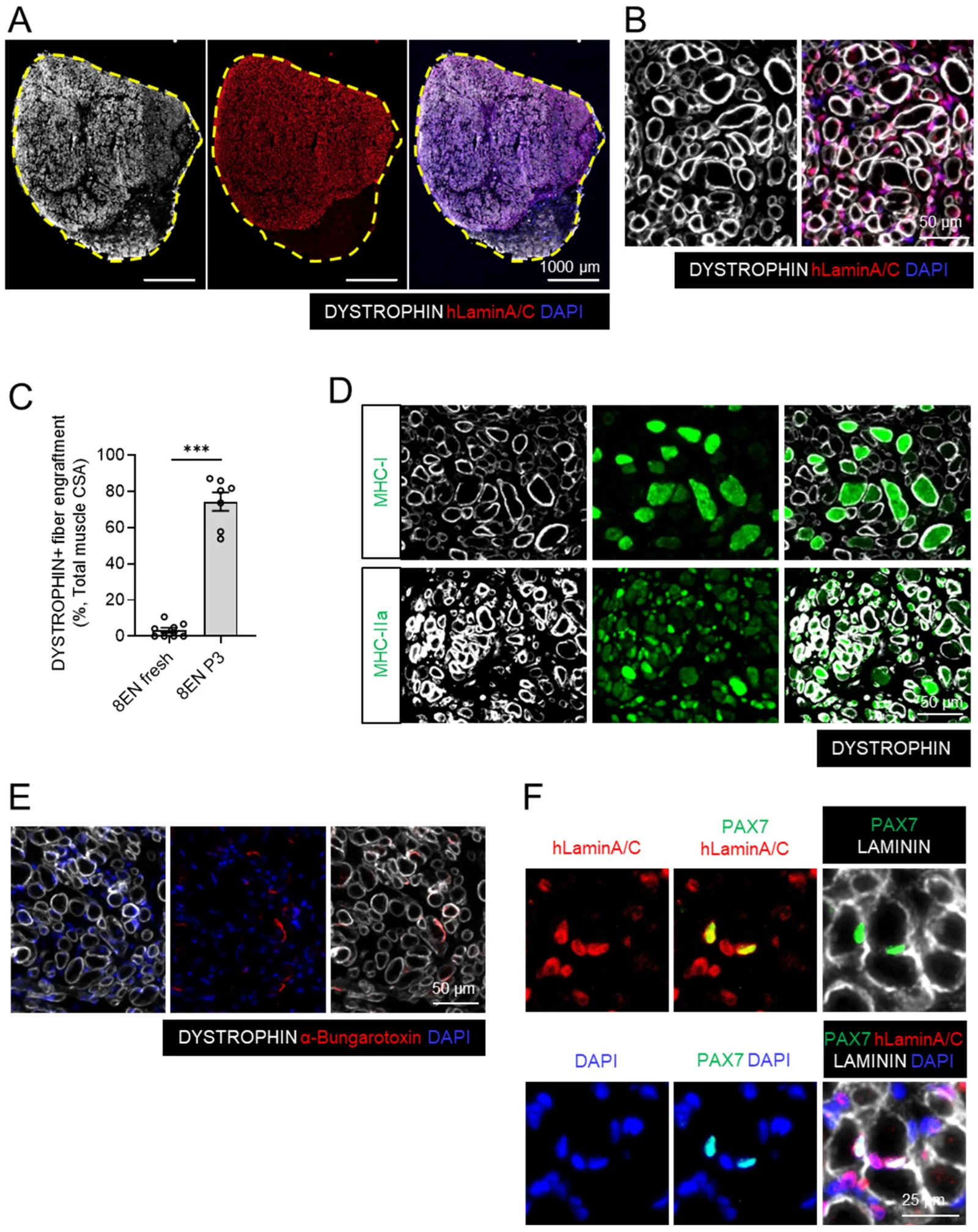
Passaged 8EN skeletal myogenic progenitors engrafted to form new muscle fibers and to reconstitute the muscle stem cell pool after transplantation. Passage 3 8EN cells engrafted and formed new dystrophin^+^ fibers: (**A**) the whole TA muscle (scale bar = 1000 μm), (**B**) magnified image (scale bar = 50 μm). Human lamin A/C expression in cell nuclei indicated human origins, i.e., donor cell-derived cells. (**C**) Quantification of fiber engraftment from freshly-isolated vs. passage 3 8EN cell transplantation (mean ± SEM, 7–8 biological replicates, *** *p* < 0.001). In both cases, 100,000 cells were transplanted. (**D**) Newly formed dystrophin^+^ fibers expressed adult myosin isoforms MHC-I and MHC-IIa (scale bar = 50 μm). (**E**) The proximity of α-bungarotoxin^+^ post-synaptic staining and dystrophin^+^ fibers suggested potential neuromuscular junctions and innervation (scale bar = 50 μm). (**F**) Transplanted passaged 8EN cells repopulated the muscle stem cell pool, as evidenced by donor cell-derived human lamin A/C^+^ PAX7^+^ cells residing in the fiber basal lamina (scale bar = 25 μm). 8EN: CD82^+^ ERBB3^+^ NGFR^+^; MHC: myosin heavy chain.

The enhanced survivability of the passaged 8EN cells encouraged us to study their muscle regeneration potential in vivo. We transplanted 100,000 passage 3 8EN cells to the TA muscles of NSG-mdx^4Cv^ mice as described above and evaluated the transplanted muscles for engraftment 3 months later. Remarkably, we observed an exceptional level of fiber engraftment—~75% of the total muscle volume was remuscularized by donor cell-derived fibers (i.e., human lamin A/C^+^ dystrophin^+^) (Figure 4A–C). This level of fiber engraftment is comparable to what we observed when we transplanted mouse teratoma-derived skeletal myogenic progenitors or endogenous adult muscle stem cells [18,19]. The newly formed fibers expressed adult MHC isoforms, including MHC-I and MHC-IIa, which might indicate that the donor cells mature in vivo (Figure 4D). We also observed positive α-bungarotoxin staining adjacent to donor-derived dystrophin^+^ fibers, suggesting the presence of neuromuscular junctions and potential innervation (Figure 4E). Moreover, we found human lamin A/C^+^ PAX7^+^ cells residing in the muscle basal lamina, implying that the donor cells repopulated the endogenous muscle stem cell pool (Figure 4F).

## 4. Discussion

Generating functional skeletal muscle cells with efficient engraftment potential from human PSCs is challenging. We have previously shown that teratoma formation is a feasible strategy for producing skeletal myogenic progenitors from human PSCs. In this study, we found that the combination of three surface proteins, CD82, ERBB3, and NGFR, marked an engraftable skeletal myogenic population from human PSC-derived teratomas. These 8EN cells were PAX7^+^/MYOD^+^, capable of forming multinucleated MHC^+^ myotubes and expandable in vitro. Importantly, upon intramuscular transplantation, the expanded cells regenerated muscle fibers and developed into PAX7^+^ cells, repopulating the endogenous muscle stem cell niche.

NGFR (nerve growth factor receptor, also known as CD271, p75NTR, and TNFRSF16) is a cell surface protein mediating signals important for neuronal development, cell survival, growth, and differentiation [27,28]. NGFR is mainly expressed in neural crest stem cells and other progenitor populations during development [29,30,31]. Recently, NGFR was found to be expressed in human skeletal myogenic cells at the fetal stage as early as week 8, and in skeletal myogenic progenitor cells derived from hPSCs by in vitro monolayer differentiation [13]. In our previous single-cell RNA-seq analysis, we found that skeletal myogenic cells from hPSC-derived teratomas were largely embryonic/fetal [20]. We further showed in this study that NGFR is a useful surface marker for enriching an engraftable skeletal myogenic population from hPSC-derived teratomas.

Donor cell survival after transplantation is essential for them to engraft. In a previous study, transplantation of a single myofiber containing ten muscle stem cells had an engraftment efficiency two-fold higher than that of transplanting an equivalent number of isolated muscle stem cells, i.e., ten muscle stem cells [3,4]. Although this result is generally interpreted as how the niche environment of the myofiber improves the engraftability of muscle stem cells, it is also possible that the additional digestion step used to isolate muscle stem cells from the residing myofibers might have damaged the former and, thus, reduced their survivability upon immediate transplantation. In this study, we used a one-hour enzyme digestion procedure to adequately isolate 8EN cells from human PSC-derived teratomas. FACS was subsequently used to sort the CD82^+^ ERBB3^+^ NGFR^+^ cell population, i.e., fresh 8EN cells. The long digestion procedure and subsequent FACS-sorting might have been stressful to cells and might have induced apoptosis, resulting in a poor survival rate of these freshly-isolated cells post-transplantation. Differently from the fresh 8EN cells, the passaged 8EN cells were not subjected to the hour-long enzyme digestion, were dissociated from tissue culture dishes within a few minutes, and did not undergo FACS-sorting before transplantation. Therefore, in comparison with fresh 8EN cells, passaged 8EN cells experienced a less stressful handling procedure immediately before transplantation. Passaged 8EN cells were minimally apoptotic and survived better in vivo, which probably contributed to their robust engraftment upon transplantation. Nevertheless, other factors besides apoptosis are likely to be involved in facilitating the engraftment of cultured 8EN cells, and further investigation is warranted.

In vitro culture/expansion of adult muscle stem cells markedly reduces their in vivo regeneration potential [4,32]. However, human teratoma-derived 8EN skeletal myogenic progenitors were expandable in vitro while still showing potent engraftment capability. CD82 is a marker for fetal and adult human muscle stem cells [25,26], and CD56 (also known as NCAM, neural cell adhesion molecule) is a well-known marker for adult human muscle stem cells [33]. We found that passaged 8EN cells maintained CD82 positivity and started to express CD56, resulting in enhanced fusion efficiency upon myotube differentiation. We reasoned that the in vitro culture might promote the maturation of these hPSC-derived myogenic cells from embryonic/fetal skeletal myogenic progenitor cells towards cells with adult muscle stem-like phenotypes, and it thereby contributes to the potent regeneration potential. It would also be interesting to examine which components in the culturing medium affected the maturation of human skeletal myogenic progenitors.

In the current study, we have demonstrated that an expandable and engraftable CD82^+^ERBB3^+^NGFR^+^ skeletal myogenic progenitor population can be produced from human PSCs via teratoma formation. Further investigations into this in vivo differentiation strategy may provide valuable insights into generating muscle stem cells from PSCs in vitro and, thereby, advance cell therapy for muscular diseases.

## Data Availability

The original contributions presented in this study are included in the article/Supplementary Materials. Further inquiries can be directed to the corresponding author.

## Supplementary Materials

The following supporting information can be downloaded at: https://www.mdpi.com/article/10.3390/cells12030362/s1, Figure S1: FACS profiling of 8EN cells over 3 passages; Figure S2: Apoptosis analysis of fresh isolated and passaged 8EN cells.
