# Supplementary material for: Efficient Muscle Regeneration by Human PSC-Derived CD82+ ERBB3+ NGFR+ Skeletal Myogenic Progenitors
Source: Cells. 2023 Jan 18;12(3):362. doi: 10.3390/cells12030362 (PMC9913306; doi:10.3390/cells12030362)
Supplement: Supplementary file 1 [file cells-12-00362-s001.zip › cells-2142884-supplementary.pdf]

Figure S1

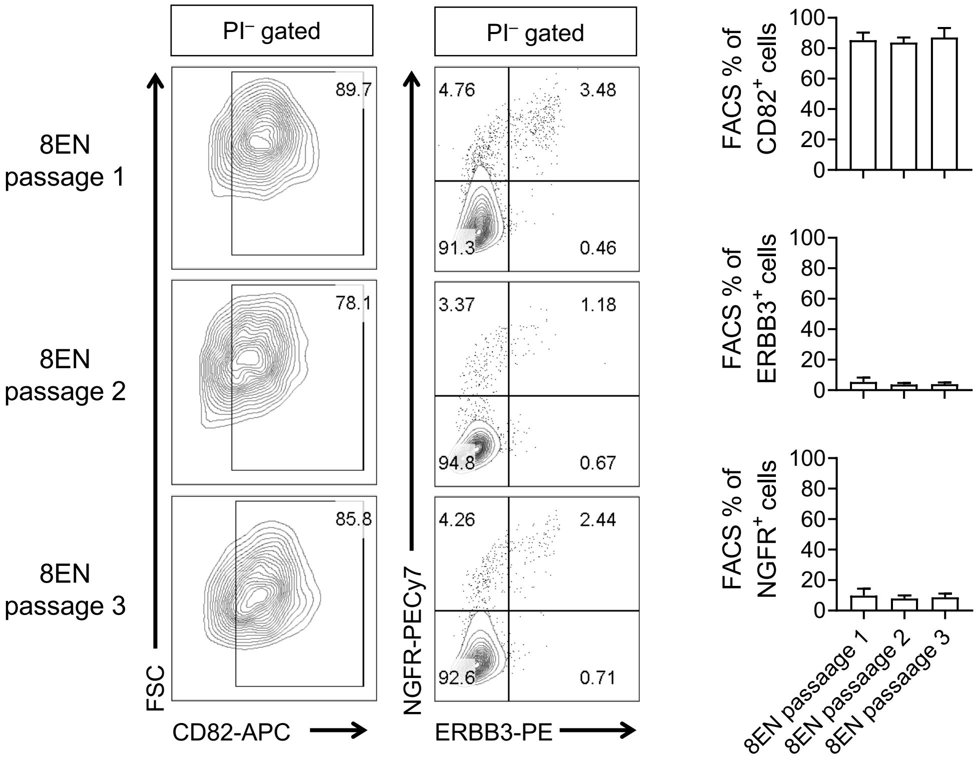

Figure S1, related to Figure 2, FACS profiling of 8EN cells over 3 passages
FACS profiling (left) and quantification (right) of CD82, ERBB3, and NGFR expression in passaged 8EN cells. Data are shown as mean ± SEM from 3 biological replicates. No statistical significance in FACS % of all 3 markers was observed among the 3 passages (one-way ANOVA). Note that the FACS plot and quantification for 8EN passage 3 cells were also used in Figure 2B.
8EN: $CD82^+$ $ERBB3^+$ $NGFR^+$.

Figure S2

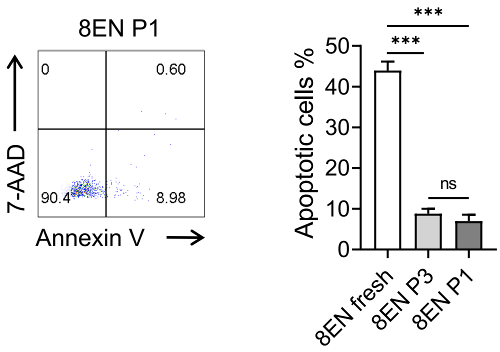

Figure S2, related to Figure 3, Apoptosis analysis of fresh isolated and passaged 8EN cells

FACS analysis (left) and quantification (right) of passage 1 8EN cells (8EN P1) for apoptosis. Cells expressing 7-AAD and/or Annexin V were considered as apoptotic. Data are shown as mean $\pm$ SEM from 4 biological replicates. ***$p < 0.001$; ns: not significant. Note that quantification for 8EN fresh and 8EN P3 were also used in Figure 3C.
8EN: $CD82^{+}$ $ERBB3^{+}$ $NGFR^{+}$.
